# An Efficient Data Compression Model Based on Spatial Clustering and Principal Component Analysis in Wireless Sensor Networks

**DOI:** 10.3390/s150819443

**Published:** 2015-08-07

**Authors:** Yihang Yin, Fengzheng Liu, Xiang Zhou, Quanzhong Li

**Affiliations:** 1 School of Data Science and Computer, Sun Yat-Sen University, Guangzhou 510006, China; E-Mails: yinyh3@mail2.sysu.edu.cn (Y.Y.); liufzh3@mail2.sysu.edu.cn (F.L.); zhoux85@mail2.sysu.edu.cn (X.Z.); 2 Collaborative Innovation Center of High Performance Computing, National University of Defense Technology, Changsha 410073, China

**Keywords:** data compression, principal component analysis, cluster, wireless sensor network

## Abstract

Wireless sensor networks (WSNs) have been widely used to monitor the environment, and sensors in WSNs are usually power constrained. Because inner-node communication consumes most of the power, efficient data compression schemes are needed to reduce the data transmission to prolong the lifetime of WSNs. In this paper, we propose an efficient data compression model to aggregate data, which is based on spatial clustering and principal component analysis (PCA). First, sensors with a strong temporal-spatial correlation are grouped into one cluster for further processing with a novel similarity measure metric. Next, sensor data in one cluster are aggregated in the cluster head sensor node, and an efficient adaptive strategy is proposed for the selection of the cluster head to conserve energy. Finally, the proposed model applies principal component analysis with an error bound guarantee to compress the data and retain the definite variance at the same time. Computer simulations show that the proposed model can greatly reduce communication and obtain a lower mean square error than other PCA-based algorithms.

## 1. Introduction

Wireless sensor networks (WSNs) consist of a great number of tiny sensor nodes that are obviously capacity constrained, especially power constrained. Generally, each sensor node has three functions, *i.e.*, sensing the requisite information, processing and managing the acquired data and exchanging messages with other sensor nodes [1]. Because inter-node communication consumes most of the power, efficient data compression schemes are used to reduce the data transmission in order to prolong the lifetime of wireless sensor networks [2].

For the purpose of conserving energy, a great deal of data aggregation models have been proposed in recent years, including principal component analysis (PCA)-based algorithms. PCA is one of the dimensionality reduction models, which uses an orthonormal transformation to convert a set of observations of possibly correlated variables into a set of values of linearly-uncorrelated variables called principal components. The number of principal components (PCs) is smaller than or equal to the number of original variables [3]. The PCA operation can be regarded as revealing the internal structure of the data in a way that best explains the variance in the data. Due to such characteristic, PCA can be used effectively to compress data in WSNs.

Recently, the PCA-based algorithms have been applied to wireless sensor networks [4,5,6,7,8,9]. In [4,5], the authors proposed a data compression model based on context that worked in an orthogonal way and utilized the attribute of each individual component to reduce the data transmission. In [6], Borgne *et al*. showed that the PCA can be efficiently implemented in a network of wireless sensors, where supervised and unsupervised compression models are presented. Following that, they revealed a distributed power iteration method to compute an approximation of the principal components in [7]. Rooshenas *et al*. [8] proposed an algorithm, which let the sink node gain access to the original data for computing the reconstruction error to get a tradeoff between the accuracy and the rate of compression. Fenxiong *et al*. [9] proposed an algorithm based on multiple-PCA, which iteratively uses the PCA method in multiple layers.

These above-mentioned PCA-based models, however, mostly ignore the strong temporal-spatial correlation and massive data redundancy among sensor nodes, which are vital for reducing data transmission and saving power consumption in WSNs. In the real world, temporal-spatial relevancy among sensor nodes usually exists. By grouping similar sensor nodes into one cluster, the interdependency of sensor data will gain a considerable improvement. Therefore, we can use less principal components to represent more original data, and the performance of the compression is expected to be significantly promoted.

A process of spatial clustering can be used to find the correlation among sensor data. Regarding the spatial clustering, the authors in [10] proposed a hierarchical spatial clustering algorithm, which aims to group the highly-correlated sensor nodes into the same cluster for rotatively reporting representative data later. In [11], Bandyopadhyay *et al*. proposed a distributed, randomized clustering algorithm to organize the sensors and generate a hierarchy of cluster heads.

These spatial clustering models are usually used for approximate data collection, where the data of the cluster head is used to represent all of the data of the same cluster. Obviously, the precision of the model will be influenced greatly by the correlation of the sensor data. It will have a poor performance, while the relevance among sensor data is not strong enough.

In this paper, we propose an efficient data compression model, which is based on spatial clustering and principal component analysis to aggregate data, reducing the transmission data while ensuring the accuracy of compression. Moreover, by using magnitude similarity to measure the current state of sensor data and the autoregressive model to capture the varying trend of the environment, our proposed model has considerable adaptability for various situations.

In addition, an adaptive cluster head selection strategy is also proposed to achieve the purpose of economizing energy. It can be regarded as an extension of the cluster head selection strategy in the DDSPalgorithm in [12], as the cluster head selection in DDSP is uncorrelated in different rounds, and they just assumed that a node autonomously decides to elect itself the cluster head with probability *p*, while our model takes the correlation of different periods into consideration. By doing that, the energy consumption of each sensor node can be reduced further.

The contributions of our work are summarized as follows.

- We propose a model based on spatial clustering and principal component analysis to compress the transmission data in wireless sensor networks, while the idea of taking the strong correlation among sensor data into consideration in the process of PCA is novel.
- We propose an adaptive strategy to guarantee the error bound of each sensor node, ensuring the precision of our compression model.
- We extend the cluster head selection strategy in [12], which can be used to reduce the energy consumption further.
- We verify the powerful performances of our proposed model through computer simulations.

The rest of this paper is organized as follows. Section 2 presents the background knowledge of our model. Section 3 proposes the cluster head selection strategy. Section 4 expounds the details of our proposed model. Section 5 evaluates the performance of the model and makes a comparison with other PCA-based algorithms. Finally, we conclude the paper in Section 6.

## 2. Background

In this section, we identify a variety of concepts and discuss the most related background to our proposed model.

### 2.1. Spatial Clustering and Autoregressive Model

In wireless sensor networks, spatial clustering is the process of grouping a set of sensors into clusters, so that sensor nodes within one cluster have higher similarity compared to one another, while being dissimilar to sensors in other clusters. Spatial clustering can be used to gain insight into the distribution of the data, to capture the underlying pattern of the cluster and to focus on a particular set of clusters for further analysis [11]. By grouping the similar sensor nodes into one cluster and aggregating the sensor data into the cluster head node, we can obtain a set of data with a strong correlation, and thus, effective algorithms can be employed to compress the data accordingly.

For spatial clustering, a key question is how to measure the similarity between the readings of any two sensor nodes. Some existing algorithms considered the magnitude similarity as the criterion, such as DClocalin [13] and DACA in [14]. However, magnitude similarity just grasps the current temporal feature of sensor data and ignores the underlying varying trend. Thus, it cannot capture the dynamic change of the environment. Some other algorithms, such as Elink [15], only relied on the trend similarity. Taking the trend similarity into consideration will overlook the benchmark of the sensor data, which causes the indications of the sensor nodes in one cluster to have little in common.

In order to avoid the above-mentioned problem, we take both magnitude similarity and trend similarity into account. For magnitude similarity, our model proposes to keep a sequence of readings and to calculate the Euclidean distance of any two sensor nodes.

As for trend similarity, the autoregressive (AR) model can be constructed for each sensor node to capture the tendency of the environmental change and to measure the trend similarities among sensor nodes. The autoregressive model can describe certain time-varying processes, which specifies that the output variable depends linearly on its own previous values. The notation AR(*n*) indicates an autoregressive model of order *n*. The AR(*n*) model is defined as:

(1) $$x_{t}=c+\sum_{i=1}^{n}\varphi_{i}x_{t-i}+\varepsilon_{t}$$

where $\varphi_{i}$ is the parameter of the model, *c* is a constant and $\varepsilon_{t}$ is the white noise. The calculation of the AR parameters is diverse, e.g., we can regard the first *n* readings as the input and the (n+1)-th reading as the output, then construct a training set in this way; then, the problem can be treated as a linear regression, and the parameters can be estimated by least squares and gradient descent.

Through sending the parameters of the AR model and a sequence of sensor readings to the sink node, sensor nodes can be classified into different clusters by designated cluster algorithms, in which the clustering process not only depends on the magnitude similarity, but the trend similarity, as well [10].

### 2.2. Principal Component Analysis

**Figure 1:**
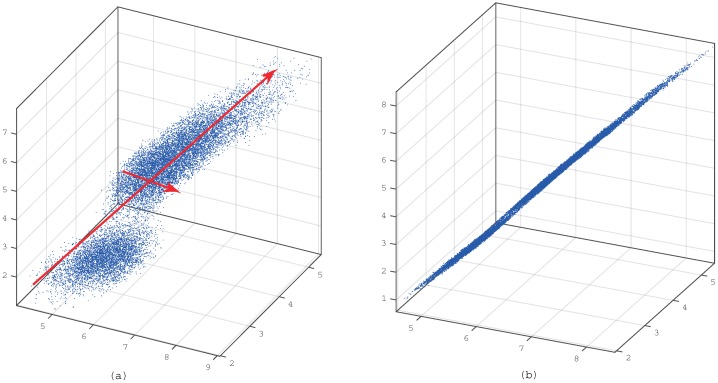
Illustration of the principal component analysis. The red line in (**a**) represents the direction of PCs. If we look at the data in the plane identified by PCA which can be seen in (**b**), it was mostly 2D, as well as keeping almost the whole of the variances.

Principal component analysis is a statistical model that projects the data onto a new basis and aims to retain variance as large as possible while minimizing the redundancy [3]. It can be realized by calculating the eigenvalues and eigenvectors of the covariance matrix (covariance matrix Σ is a matrix whose element denotes the tendency of jointly varying; assume $X_{i}$ and $X_{j}$ are random scalars, then their covariance can be calculated by $\Sigma_{ij}=cov\left(X_{i},X_{j}\right)=E\left[\left(X_{i}-E\left(X_{i}\right)\right)\left(X_{j}-E\left(X_{j}\right)\right)\right]$) of the data. Once eigenvectors are sorted by the homologous eigenvalue in descending order, the eigenvectors denote principal components, and the one corresponding to the maximum eigenvalue relates to the dominant principal component. Then, a transformation matrix can be constructed with the first definite number of eigenvectors to project the data onto a new basis. Suppose $X_{m\times n}$ denotes the original matrix, $W=(w_{1},w_{2},\cdots ,w_{n})$ represents the eigenvectors of the covariance matrix of *X* and $w_{i}$ represents a column vector of the covariance matrix *W*. The transformation matrix Θ can be constructed by $\Theta =\left(\theta_{1},\theta_{2},\cdots ,\theta_{p}\right)=\left(w_{1},w_{2},\cdots ,w_{p}\right)$(p≤n), and an approximation of the original matrix can be calculated by:

(2) $$\hat{X}=\Theta \Theta^{T}X=\Theta Z$$

where:

(3) $$Z=\Theta^{T}X$$

represents the projection of the original data onto the principal components' base. The process of calculating the principal components can be regarded as minimizing the optimization function:

(4) $$\begin{array}{cc} J_{p}\left(x_{i},\theta_{i}\right) & =\frac{1}{m}\sum_{i=1}^{m}{∥x_{i}-\hat{x_{i}}∥}^{2}\\ & =\frac{1}{m}\sum_{i=1}^{m}{∥x_{i}-\sum_{i=1}^{p}\theta_{i}\theta_{i}^{T}x_{i}∥}^{2} \end{array}$$

by the constraint that each $\theta_{i}$ is orthonormal, $x_{i}$ and $\theta_{i}$ represent the column vector of *X* and Θ, *m* is the number of observations and *p* is the number of principal components. A sequence of *θ* that minimizes the optimization function is the first *p* eigenvectors, which are ordered by the eigenvalues of the covariance matrix [16]. Figure 1 (following the idea from [17], we plot the figure according to our data) is an illustration of the principal component analysis by projecting the three-dimensional (Figure 1a) data onto the first two principal components' basis (Figure 1b). It is clear that the PCA keeps the direction of the first two maximum variances [18].

The ratio of retained variance after transforming by *p* principal components can be measured by:

(5) $$\begin{array}{c} R\left(p\right)=\frac{\sum_{i=1}^{p}\lambda_{i}}{\sum_{i=1}^{n}\lambda_{i}} \end{array}$$

$\lambda_{i}$ is the eigenvalue of the covariance matrix. R(p) can be considered as a metric to evaluate the accuracy of compressing.

## 3. The Cluster Head Selection Strategy

In this section, we propose a cluster head selection strategy based on the first order radio model [19] to conserve energy and prolong the lifetime of wireless sensor networks. The inspiration comes from heuristic searching [20].

**Figure 2:**
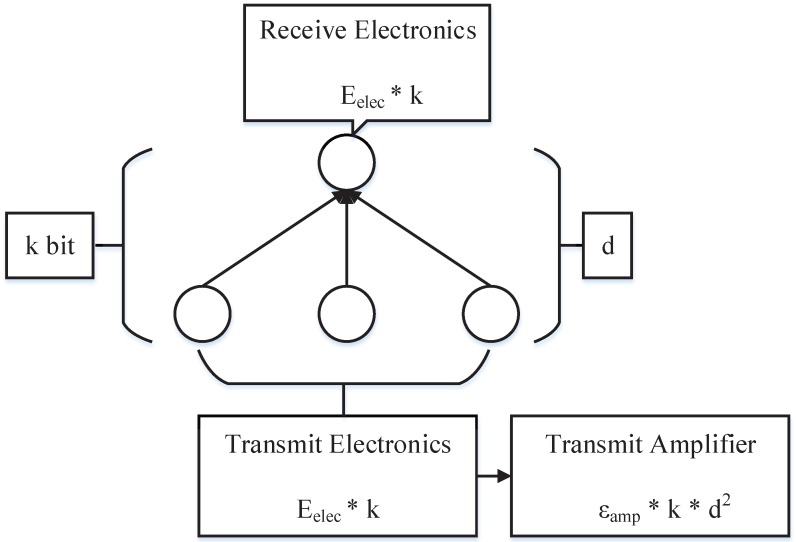
The first order radio model.

An illustration of the first order radio model is shown in Figure 2. The energy consumption of transmitting a *k*-bit packet at a distance *d* can be expressed by:

(6) $$E_{Tx}\left(k,d\right)=\left\{\begin{array}{cc} E_{elec}*k+\varepsilon_{amp}*k*d^{2}, & \text{if}\;d<d_{0}\\ E_{elec}*k+\varepsilon_{amp}*k*d^{4}, & \text{if}\;d>=d_{0} \end{array}\right.$$

and receiving a *k*-bit packet can be calculated by:

(7) $$\begin{array}{c} E_{Rx}\left(k\right)=E_{elec}*k \end{array}$$

where $E_{elec}$ is the radio dissipation of running the electric circuit to transmit or receive a message, $\varepsilon_{amp}$ is used for the transmit amplifier to ensure the smooth operation of the radio, *k* is the size of the transmitting or receiving packet, *d* is the distance between the transmission node and receiver node and $d_{0}$ is a predefined value, which depends on the performance of the sensor node.

In our model, as a transmitting node, the energy consumption can be expressed by $E_{Tx}\left(k,d_{1}\right)$, where $d_{1}$ is the distance between the current sensor node and the cluster head node. As the cluster head node, it will receive packets from other sensor nodes in the same cluster and transmit all of the packets to the sink node, which will expend:

(8) $$\begin{array}{c} E_{consumption}=E_{Rx}\left(k\right)*\left(n-1\right)+E_{Tx}\left(k*n,d_{2}\right) \end{array}$$

where we suppose all sensor nodes transmit a *k*-bit packet for simplicity, *n* is the number of nodes in current cluster and $d_{2}$ denotes the distance between the cluster head node and the sink node.

After each clustering procedure, we calculate the energy consumption $E_{consumption}$ of each node for each cluster and then select the node with the lowest $E_{consumption}$ as the head of current cluster, which can be expressed by:

(9) $$\begin{array}{c} C_{head}^{\left(i\right)}=\arg\min_{\left\{n_{j}^{\left(i\right)}\right\}}\left(E_{consumption}\left(n_{j}^{\left(i\right)}\right)\right) \end{array}$$

$n_{j}^{\left(i\right)}$ indicates that sensor node *j* belongs to the *i*-th cluster, $C_{head}^{\left(i\right)}$ represents the head of the *i*-th cluster, and it is the sensor node that is in the *i*-th cluster that minimizes $E_{consumption}$. In order to scatter the power expenditure over all of the sensor nodes, we also propose a rotatory strategy to select the cluster head. $E_{past}\left(k\right)$ is used to record the total power consumption of the *k*-th sensor node, and $E_{consumption}\left(k\right)$ represents the consumption of the *k*-th node in the context of the *k*-th node being the head of the current cluster; then:

(10) $$\begin{array}{c} C_{head,\;rotatory}^{\left(i\right)}=\arg\min_{\left\{n_{k}^{\left(i\right)}\right\}}\left(E_{past}\left(n_{k}^{\left(i\right)}\right)+E_{consumption}\left(n_{k}^{\left(i\right)}\right)\right) \end{array}$$

can be used to select the head of each cluster. After each decision epoch, the consumption of the *k*-th node at the current epoch is added to $E_{past}\left(k\right)$, whose value is $E_{Tx}\left(k,d_{1}\right)$ for the member of the cluster or $E_{consumption}$ for the head of the cluster.

## 4. System Model

In this section, we formalize the compression procedure and summarize the system model.

### 4.1. Notations and Formalization

In this paper, we consider a wireless sensor network that consists of a set $S=\{s_{1},s_{2},\cdots ,s_{m}\}$ of *m* sensor nodes and one sink node. All of the sensor nodes are distributed randomly in a region. Sensor nodes acquire a sequence of data by epoch, which is a discrete time domain where sensor readings are gleaned and notated by T={1,2,⋯n}.

Suppose $x_{i}\left[t\right]$ denotes the sensor node *i* at epoch t∈T and $X\left[t\right]=(x_{1}\left[t\right],x_{2}\left[t\right],\cdots ,x_{n}\left[t\right])$ represents all of the sensor node readings at epoch *t*. $X_{m\times n}$ is a reading matrix that consists of *m* sensor observations at *n* epochs and whose elements $x_{ij}=x_{i}\left[t\right]$, i<=m and j<=n.

Now, we can apply principal component analysis to the sensor data matrix $X_{m\times n}$, which can be obtained at the cluster head node, with the goal to find an orthonormal matrix *W* to construct the transformation matrix Θ, transforming the data matrix into a new space according to Equation (3). Then, $Z_{m\times p}$ that we gained by the transformation is sent to the sink node, where *p* is the number of principal components and p<n. Here, we use an adaptive strategy to decide the value of *p* for guaranteeing the error bound of each node and roughly constraining the accuracy of the overall model, by which the data matrix has been compressed in a certain proportion. The transformation matrix Θ, which can be calculated at the cluster head node, will be sent together with *Z* to the sink node in order to reconstruct the data matrix.

### 4.2. Compression Model

**Figure 3:**
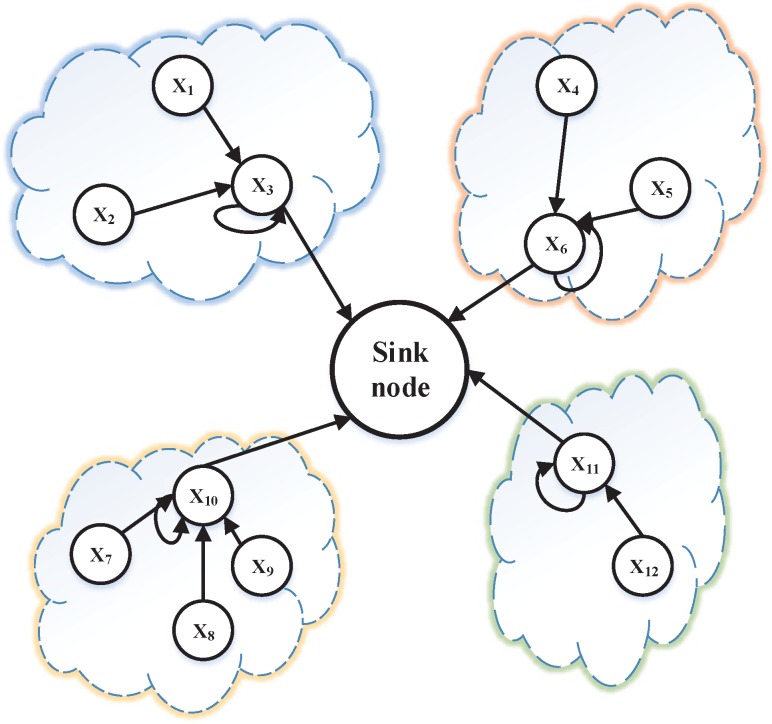
Diagrammatic sketch of our model.

A simple diagrammatic sketch of our model is illustrated in Figure 3, where nodes being in one case represents that they are classified into one cluster through the similarity measure metric, which we defined in Section 2.1, and the cluster head nodes gather data of all nodes in their own cluster and handle the data by the predefined compression algorithm.

**Figure 4:**
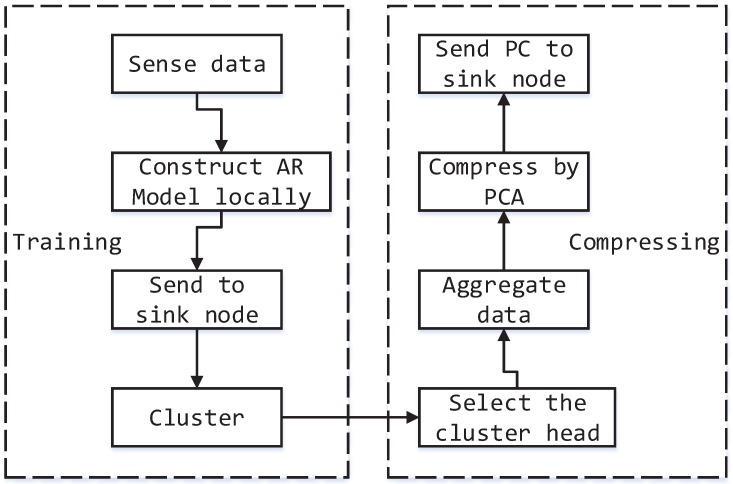
Execution procedure of our model.

The holistic compression procedure can be seen in Figure 4.

This procedure consists of two periods, *i.e.*, training by collecting the historical data to discover the underlying temporal-spatial correlation between sensor nodes and compressing the data based on the relevance found by training. It is illustrated in detail as follows:

- First, a set of historical data of each sensor is collected and processed into a matrix by the method proposed in Section 2.1, *i.e.*, using the latest (n−1) readings as the input and the *n*-th data as the output. The *n* readings can be regarded as an observation, and *m* observations are obtained in the same way. The data of each sensor can be represented by a matrix of size m×n.
- Then, to avoid the cost of transmitting data to the sink node, we construct an autoregressive (AR) model for each sensor locally based on the data matrix acquired by the above process. The learning phase of the AR model can be considered as a linear regression, which is universal in machine learning. The method of minimizing the mean square error between the real data and predicted data can be used to estimate the parameter through gradient descent. For more details, refer to [21].
- Next, the AR parameters and a sequence of sensor readings of each node will be sent to the sink node. The power cost of this transmitting can be ignored, because the temporal-spatial correlation will not change frequently, and the relevancy needs to be updated at a long interval. After all of the data has been gathered into the sink node, we use a clustering algorithm to group the sensor nodes into different clusters [22]. The process of clustering can discover the underlying pattern and correlation of different sensor nodes. In our model, we use the k-means clustering algorithm, which aims to partition the *m* observations into *k* collections $C=\{c_{1},c_{2},\cdots ,c_{k}\}$, so as to minimize the sum of the distance between samples and the corresponding cluster centroid, which can be formalized by:
  (11) $$\arg\min_{C}\sum_{i=1}^{k}\sum_{x\in c_{i}}{∥x-u_{i}∥}^{2}$$
  where $u_{i}$ is the mean of points in $c_{i}$. Now, the training stage has come to an end.
- Further, the result of clustering will be distributed to each sensor node, and the correlation between sensor nodes has been clear and definite. Then, the cluster head selection strategy mentioned in Section 3 will be used at each transmission epoch to ensure the head of each cluster, and all if the data of each cluster will be gathered into the cluster head and then compressed by principal component analysis, *i.e*., each sensor node transmits a *k*-bit message synchronously. Suppose that there are *m* sensor nodes in the current cluster, then the head of the current cluster will get a $X_{m\times k}$ data matrix. Accordingly, we can get the covariance matrix $\Sigma_{k\times k}$ through the equation:
  (12) $$\Sigma =E[{\left(X-E\left[X\right]\right)}^{T}\left(X-E\left[X\right]\right)]$$
  The eigenvector matrix $W_{k\times k}$ of the covariance matrix Σ can be calculated through the eigenvalue decomposition. Following this, we can get the transformation matrix $\Theta_{k\times p}$ based on the number p(p<k) of PCs by selecting the first *p* columns of the eigenvector matrix *W*. The value of *p* can be decided by Equation (5) to guarantee the error bound of our model. To elaborate, we calculate the R(p) at each cluster head node and set *p* to the minimum value that satisfies the inequation R(p)>δ, where *δ* is a predefined value to measure the error bound that the system can tolerate. Afterwards the data matrix can be transformed into a new space by $Z_{m\times p}=X\Theta$ (The formula is a little different from Equation (3), just owing to the difference between the form of the original data expression. In Equation (3), observations are arranged by columns; however, they are arranged by rows here). Due to the strong temporal-spatial correlation between different nodes in the same cluster, we can use fewer PCs to transform the original data while retaining a considerable variance. Thus, the goal of compressing will come true at a lower cost.
- Finally, the data matrix after compression $Z_{m\times p}$ and the transformation matrix $\Theta_{k\times p}$ will be sent to the sink node, and the data matrix can be reconstructed at the sink node by $\hat{X}=Z\Theta^{T}$. Thereafter, we can calculate the mean square reconstruction error to evaluate the accuracy of the compression model, which can be used for the reference of tuning parameters.

Regarding the complexity of our proposed model, the computational parts consist of the process of clustering and compressing. For spatial clustering, we choose the Lloyds k-means algorithm, and its computational complexity is often given as O(nkdi), where *n* is the number of *d*-dimensional vectors, *k* the number of clusters and *i* the number of iterations needed until convergence. For the process of compression, the complexity of principal component analysis can be shown as $O(p^{2}m+p^{3})$ in which the covariance matrix computation is $O(p^{2}m)$ and the eigenvalue decomposition is $O(p^{3})$; *p* is the number of principal components, and *m* is the number of observations. Thus, the computational complexity of our model can be regarded as $O(nkdi+p^{2}m+p^{3})$.

### 4.3. Cluster Maintenance

As the surroundings monitored by sensor nodes constantly change, the correlation among cluster members may vary with time. The relevancy ensured by the training stage may not hold any more after a period of time, so the cluster relation needs adaptive maintenance [23]. Whenever the mean square reconstruction error has a significant increase exceeding the threshold and the retained variance has an obvious decrease, while other conditions remain unchanged, it is reasonable to suspect that the correlation among sensor nodes has changed. Thus, the clustering should be updated to keep the accuracy of compression model. It can be ordinarily realized by rerunning the training stage mentioned in Section 4.2.

## 5. Performance Evaluation

In this section, a simulation experiment has been executed to evaluate the performances of the proposed model. Moreover, we also make a comparison with two existing PCA-based algorithms in terms of compression accuracy and power efficiency.

### 5.1. Data

**Figure 5:**
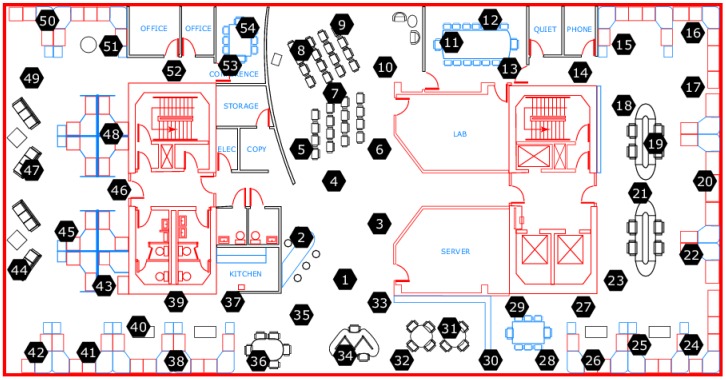
The topology structure of deployed sensors.

Data collected from 54 sensors deployed in the Intel Berkeley Research lab between 28 February and 5 April in 2004 [24] have been used to perform the experiment. The records, such as timestamped topology information, humidity, temperature, light and voltage, are collected by Mica2Dot sensors with weather boards once every 31 s. The topology information of the sensors deployed in the research lab is illustrated in Figure 5.

In the Figure 5, each sensor has a coordinate according to the distance relative to the upper right corner of the lab.

In our experiment, we choose the temperature data between 1 and 10 March of each sensor and suppose that the coordinate of the sink node is just the mean of all sensor nodes for simplicity. The missing data in some epochs are substituted by the following readings of the corresponding sensor during data preprocessing for continuity.

**Figure 6:**
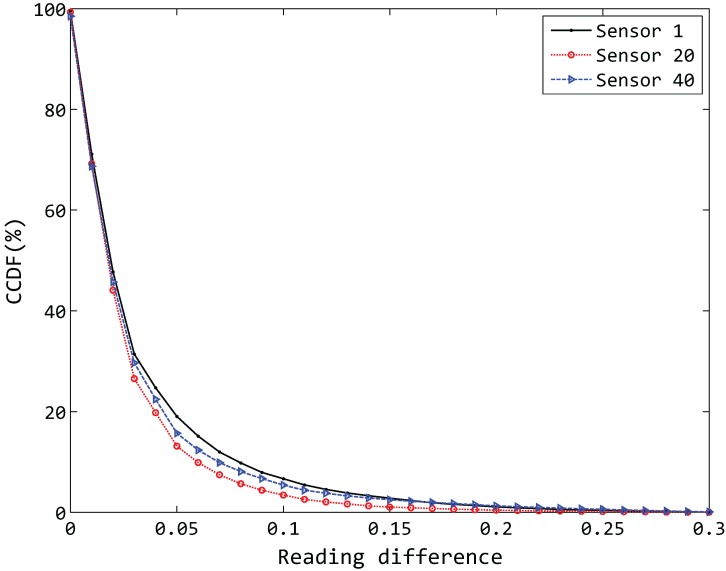
Temporal correlation in experimental data.

It is shown that the environment data in the real world at two consecutive times has a high degree of similarity, normally referred to as temporal correlation. Here, we calculate the reading difference of any two consecutive times of three different sensor nodes of our experimental data and respectively plot the CCDF (complementary cumulative distribution function) [10] in Figure 6. The value of the *y* axis is the percentage of the reading difference that is more than the current corresponding *x* axis value, e.g., the value of x=0.05 corresponds to the fact that the reading difference exceeds 0.05. Strong temporal correlation can be observed in our experimental data, as less than a 10% reading difference is greater than 0.1.

**Figure 7:**
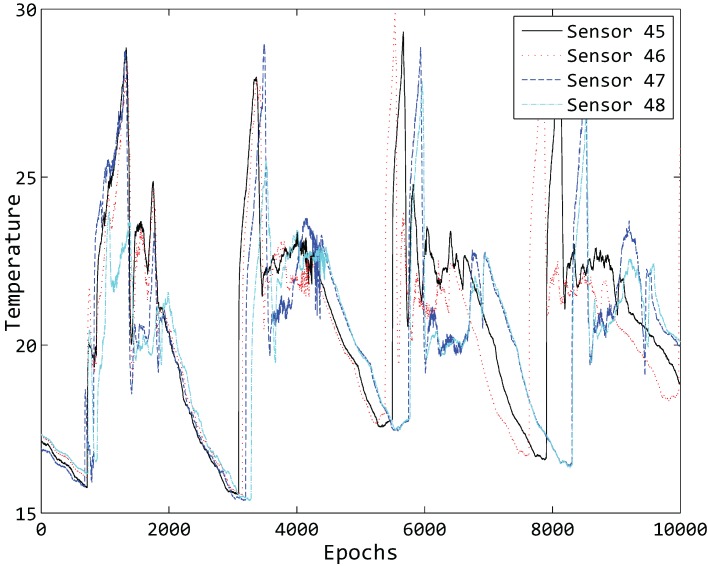
Ten thousand epochs' temperature of four different sensor nodes.

Spatial correlation usually refers to the fact that considerable similarity can be seen in the readings of neighboring sensor nodes. We plot the readings of 10,000 epochs of four different sensor nodes in Figure 7. By observing the topology structure of the deployed sensors in Figure 5, it is clear that neighboring nodes tend to obtain similar readings, showing the strong spatial correlation in our experimental data [25].

### 5.2. Parameters Setting

For the construction of an AR model of each sensor, we construct a 1000×50-sized training sample set to achieve the linear regression. It can be understood by 1000 observations and 50 epochs contained in each observation, so an AR model of 50 parameters will be acquired for each sensor. In the process of linear regression, the batch gradient descent algorithm is used to minimize the cost function, which can be formulized by the mean square error between real output and predicted output, and the parameters will be updated after each iteration until the cost function converges. In our experiment, we set the number of iterations to iter=5000 and the footstep of each iteration to α=0.00001 to ensure that the cost function can converge to the optimal value; see Table 1.

**Table 1:** Parameters used in constructing the AR model.

Parameter	Value
Number of AR model parameters	50
Size of the training sample set	1000×50
Number of iterations in batch gradient descent	5000
Footstep of each iteration	0.00001

Table 1 summarizes the parameters that are used in constructing the AR model for each sensor.

For the first order radio model mentioned in Section 3, we set the energy dissipation of receiving and transmitting to $E_{elec}=50$ nJ/bit and the radio amplifier to $\varepsilon_{amp}$ = 100 pJ/bit/m^2^. We also suppose that each transmission packet includes a 1000-bit message and the radio range of sensor nodes as $d_{0}$ = 10 m. The energy consumption of nodes whose transmission distance exceeds the predefined $d_{0}$ will be penalized by Equation 6. Note that there are different hypotheses about the radio feature. For instance, energy dissipation in the transmission process may produce a different result. The parameters setting can be seen in Table 2.

**Table 2:** Parameters used in the first order radio model.

Parameter	Value
Energy dissipation	$E_{elec}=50$ nJ/bit
Radio amplifier	$\varepsilon_{amp}=100$ pJ/bit/m${}^{2}$
Number of bits in each packet	1000 bits
Radio range of sensor nodes	10 m

For the clustering procedure, the k-means algorithm is used to group the sensor data into different clusters, and we use the squared Euclidean measure as the metric and the k-means++ algorithm for centroid initialization. More details are in [26].

The localPCA algorithm in [8] and the multi-PCA algorithm in [9] are selected as a comparison model, where a minimum-hop route method is used to construct the data collecting tree. Put simply, each node builds a minimum-hop route to the sink, so as to decide their parent node in the tree. Other existing algorithms for constructing maximum lifetime data gathering tree can also be used, such as [27]. In particular, the multi-PCA algorithm in [9] compresses the data by iteratively using the PCA method in multiple layers, which is similar to our model, but without the clustering process, so it can be used to evaluate the effect of the cluster selection in our proposed method. The iPC3algorithm in [4] and the PC3 algorithm in [5] are not considered as comparison models, as the assumption in their method is quite different from our model, which makes it difficult to have a fair comparison.

### 5.3. Compression Accuracy

Compressed data can be reconstructed at the sink node with the compressed data matrix *Z* and transformation matrix Θ by Equation (2). We use the mean square error (MSE):

(13) $$MSE=\frac{1}{m}\sum_{i=1}^{m}\frac{1}{n}\sum_{t=1}^{n}{\left(x_{i}\left[t\right]-{\hat{x}}_{i}\left[t\right]\right)}^{2}$$

as the measure metric of compression accuracy, where *m* is the number of sensor nodes and *n* is the number of epochs. The square error of each epoch $x_{i}\left[t\right]$ is summed, and the average value is calculated as the error of sensor $X_{i}$; then, the mean value of all $X_{i}$ will be considered as the mean square error of the model.

**Figure 8:**
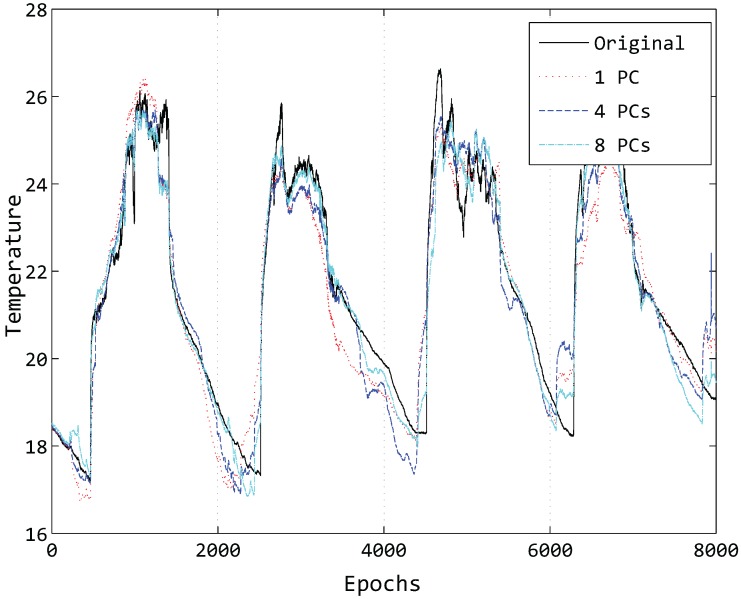
Compressed data of Sensor 1 by different PCs.

Just taking one sensor into account to think about what factors affect the compression accuracy of our model, it is obvious that the number of principal components has a great influence. An illustration of the influence of different principal components can be seen in Figure 8.

In Figure 8, we compress the temperature data of 8000 epochs of Sensor 1 using a different number of PCs and then reconstruct the data at the sink node. While the number of clusters *k* is also a key factor, here, we just set k=6 for simplicity. It can be easily found that the number of PCs is larger and that the reconstructed data curve is closer to the original data curve with a fixed value of *k* in most cases. Hence, the number of principal components is a critical factor in compressing the data and maintaining the accuracy.

Due to the fact that the accuracy can be measured by the mean square error, we calculate the mean square error of Sensor 1 from 1000 epochs to 8000 epochs by part of Equation (13), *i.e.*,

(14) $$Error_{\left(i\right)}=\frac{1}{n}\sum_{t=1}^{n}{\left(x_{i}\left[t\right]-{\hat{x}}_{i}\left[t\right]\right)}^{2}$$

**Figure 9:**
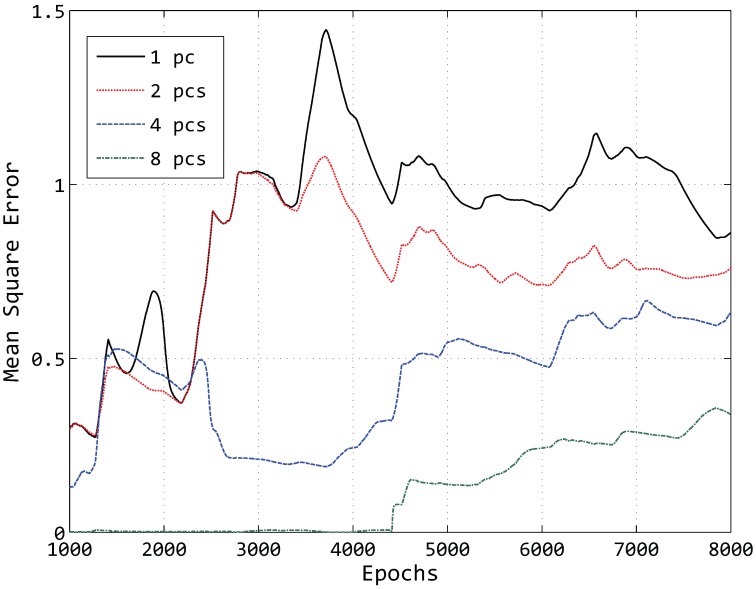
Mean square error of reconstructed data of Sensor 1.

The result is shown in Figure 9.

In Figure 9, we can see that the mean square error is decreasing with the number of principal components increasing. In addition to the number of principal components, the number of epochs is another key factor. The holistic trend of the mean square error is increasing as the number of epochs grows, since in the situation of fewer epochs, the same number of principal components can retain more variance. Taking the green line as an example, the mean square error is close to zero because eight PCs can hold almost the whole variance of the original data at the first 4000 epochs. With the increase of the number of epochs, eight PCs cannot retain the whole variance any longer. As a result, the mean square error becomes larger.

**Figure 10:**
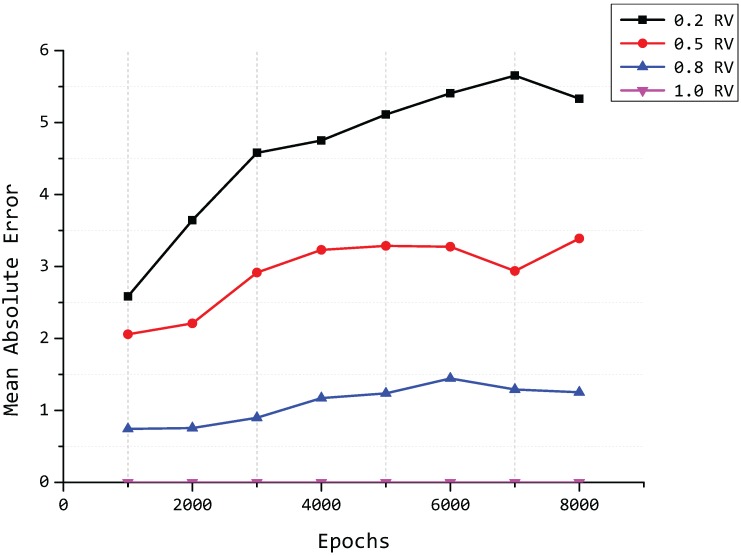
Mean square error for different values of *δ*. RV, retained variance.

As we mentioned in Section 4.2, the number of principal components is adaptively decided by the retained variance R(p) at each cluster head node, and *p* is the minimum value that satisfies R(p)>δ when we set the error bound to *δ*. Thereby, the value of *δ* is also an important factor that influences the mean square error of the proposed model. An illustration is shown in Figure 10, where retained variance (denoted as “RV” in the legend) is represented by the value of *δ*. We take eight different numbers of epochs from 1000 to 8000 and four different values of *δ* into account and then compute the mean square error in each situation. This can be seen from Figure 10.

The value of MSE is decreasing with the value of *δ* increasing, and the data are almost lossless when we set *δ* to one.

**Figure 11:**
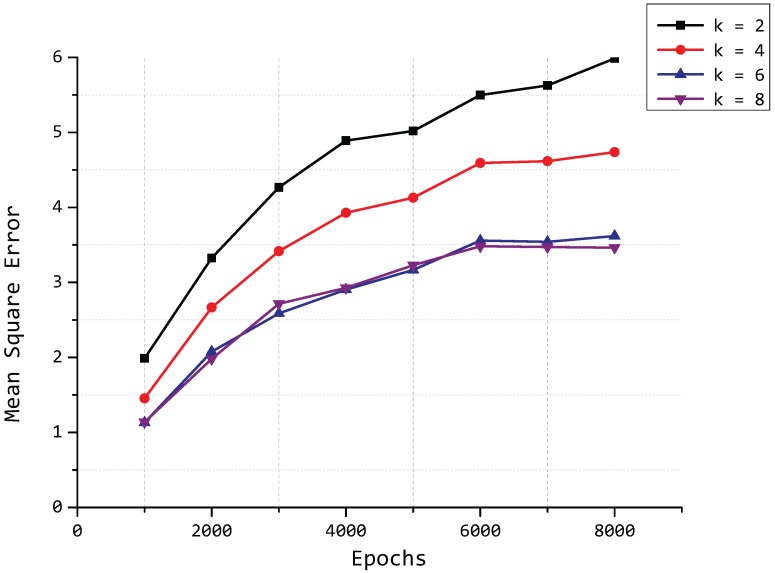
Mean square error for different values of *k*.

In addition to the values of *p* and *δ*, the number of clusters *k* also affects the compression accuracy of our model. With a fixed number of principal components p=2, we compute the mean square error of our model in a similar situation as *δ*, in which we consider eight different numbers of epochs from 1000 to 8000 and four different values of *k*. The result is plotted in Figure 11, and it can be seen that the MSE is decreasing with the increase of the value of *k*. However, we can see that the value of the MSE tends to remain stable because the fixed number of PCs can hold almost all of the variance when the value of *k* exceeds a certain value; the lines of k=6 and k=8 approach each other in Figure 11.

**Figure 12:**
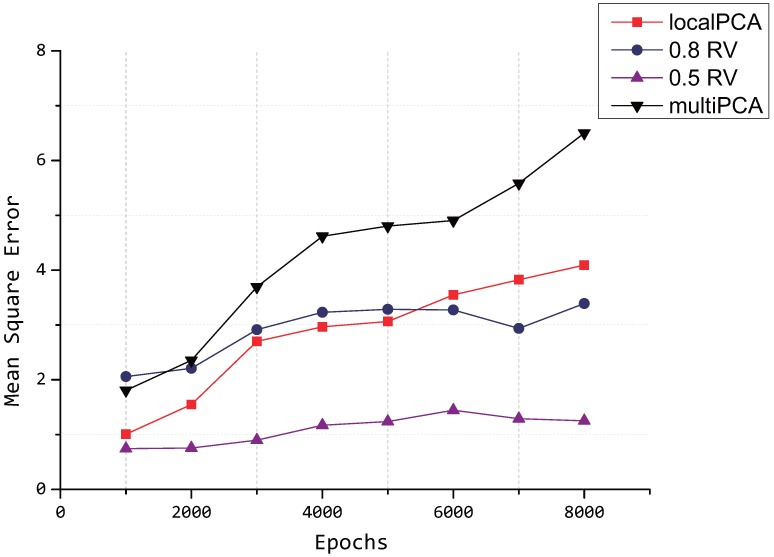
Mean square error by different algorithms.

From Figure 12, we can see that the performances of our proposed model outperforms the multi-PCA algorithm proposed in [9] and the localPCA algorithm proposed in [8], where δ=0.8. The compression accuracy of our proposed model is also better than the multi-PCA algorithm proposed in [9] when *δ* = 0.5, and it is clear that the cluster selection affects the reconstruct error.

We compare our proposed model with another two PCA-based algorithms in terms of the mean square error, which can be seen in Figure 12.

### 5.4. Compression Ratio

**Figure 13:**
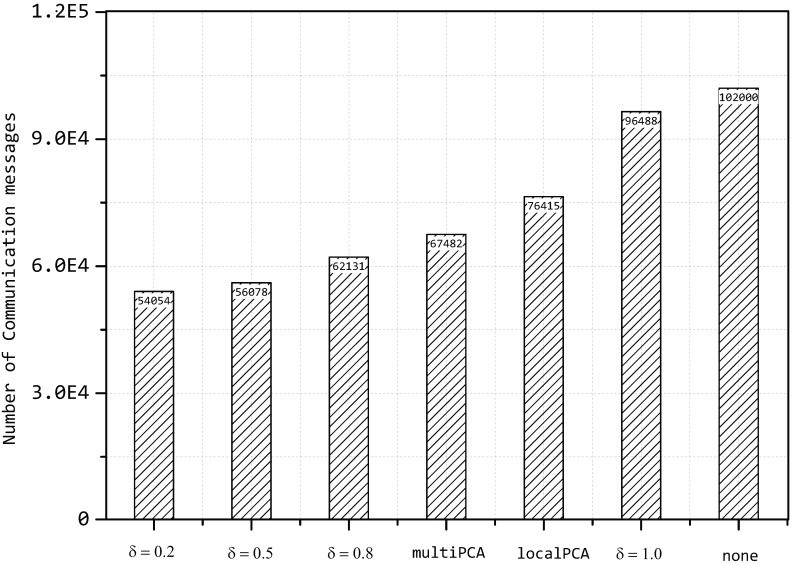
Number of communication messages with different hypotheses.

In terms of the compression ratio, we choose the number of communication messages as the measure metric, which can be used to elementarily evaluate the compression performance of our model by comparing to other algorithms. In our experiment, each sensor node sends the data to the sink node periodically involving a packet of 1000 bits each period. Due to the compression ratio of each sensor being mainly decided by R(p), there is no doubt that the number of communication messages will be severely affected by the value of *δ*. An experimental result is shown in Figure 13.

In Figure 13, the number of communication messages is changing with the value of *δ* with a fixed number of clusters k=6.

Although the transmission data will be a little less than our model if we send the data directly to the base station at each sensor node, the cost of energy consumption is so considerable that the method is not suitable for practice applications. A majority of communication messages are transmitted before compression and will not change with the value of *δ*, since the compression is implemented at the head node of each cluster.

The compression performance of our proposed model is also compared to another two PCA-based algorithms and the original method without any compression. The amount of transmission is calculated for just one period, and the result is described in Figure 13, where the performances of our proposed model are better than others, even when we set δ=0.8. From Figure 13, the gap between our proposed model and other two PCA-based algorithms for the number of communication messages grows when *δ* decreases.

According to the comparison of our model and the original method without any compression, the compression performance can be seen clearly. Additionally, comparing to the multiPCA method, it can be easily found that the cluster selection plays an important role in our model.

**Figure 14:**
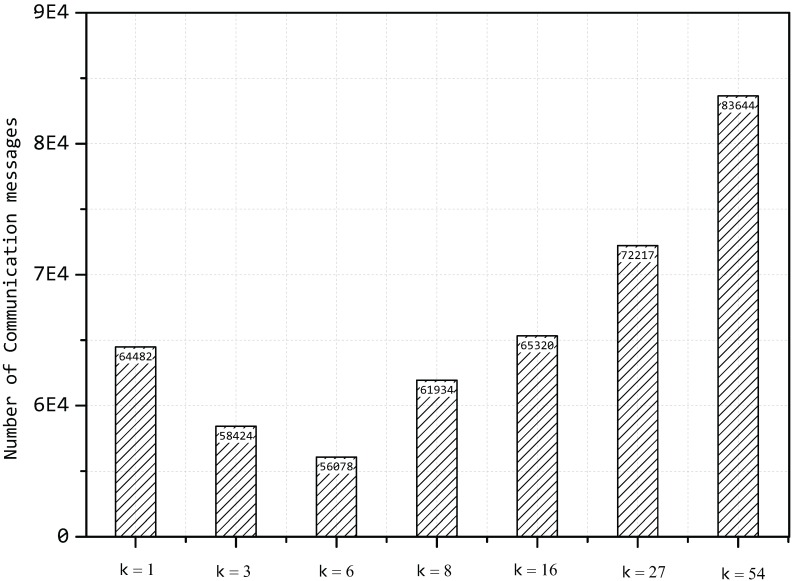
Number of communication messages with different values of *k*.

Then, we compute the number of communication messages with different values of *k* and a fixed value of δ=0.5; the result can be seen in Figure 14.

The situation of k=1 is equal to the multiPCA algorithm, and k=54 is similar to the original method without any compression; however, the number of communication messages is less than the value in Figure 13 due to the fact that we set δ=0.5 here and that sensor nodes do not need to transmit all of the data. What is more, it can be seen that the number of communication messages decreases first with the increase of the value of *k*, but increasing with a high speed when the value of *k* exceeds a certain value, since the principal components constantly vary, to retain the fixed value of *δ*. In practice, the value of *k* can be decided through a process of cross-validation, which is popular in machine learning.

### 5.5. Energy Efficiency

In terms of the energy efficiency, first, we evaluate the performances of the cluster head selection strategy proposed in Section 3 based on the first order radio model. Suppose the lifetime of the wireless sensor networks is the time when the first node in the network runs out of its energy. We set the initial power of each sensor node to $1.0\times 10^{9}\;J$ and run the transmitting process periodically until one node uses up the power. Accordingly, the number of periods representing the times of transmitting is recorded. As shown in Table 3, our proposed model can sustain 14 periods of transmitting and compressing, while multi-PCA just holds nine periods. The same model, which just replaces the cluster head selection strategy with DDSP, can sustain 11 periods, proving the effectiveness of our proposed strategy. Additionally, the model based on the multi-hop route tree can hold seven periods.

**Table 3:** Lifetime comparison with the first order radio model.

Model	Periods
Our proposed model	14
Same model with DDSPcluster head selection strategy	11
Multi-PCA model	9
Based on a multi-hop route tree	7

**Figure 15:**
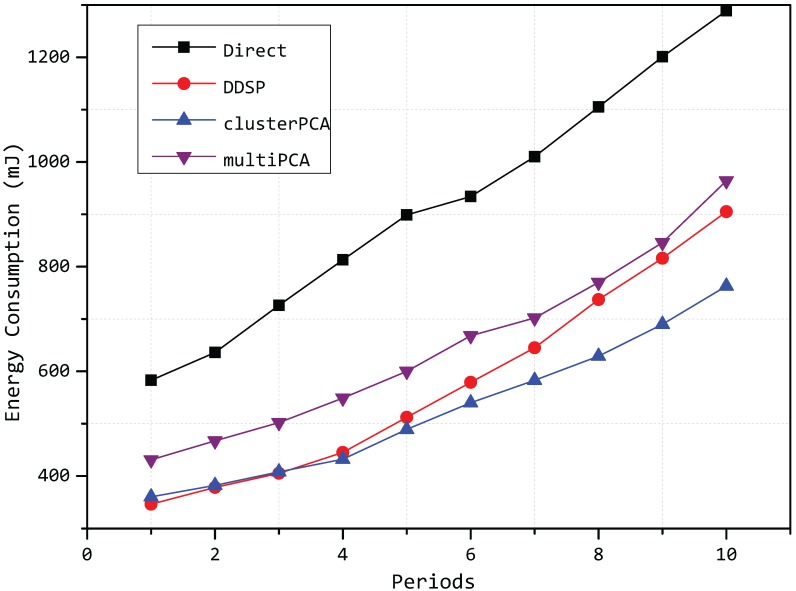
Energy consumption of each period.

Then, we compare the energy consumption of our model with other algorithms, and the parameter settings is the same as above. The DDSP algorithm in [12] is put into a comparison model because it is also a model based on a clustered architecture, to reduce the energy consumption. The total energy consumption of each period is plotted in Figure 15.

In Figure 15, we can see that the energy consumption of our model is less than the DDSP algorithm in [12], as the cluster head selection in DDSP is uncorrelated in different rounds, while our model takes the correlation of different periods into consideration. The energy consumption of our model is also less than the multi-PCA algorithm, where we can conclude the effects of cluster selection. By grouping the data into different clusters, the similarity in each cluster is considerably promoted. Through a process of PCA compression at each cluster head node, less components can be used to represent the greater variance of the original data, and the effect becomes more obvious with the periods increasing. In this way, the number of communication messages reduces greatly, and following that, the energy consumption is significantly decreased. According to the comparison, our model is more effective and efficient.

## 6. Conclusions and Future Work

Taking the spatial correlation among sensor nodes into consideration, we group the sensor nodes into different clusters according to magnitude similarity, as well as trend similarity. To conserve energy and to prolong the lifetime of wireless sensor networks, we design an adaptive cluster head selection strategy, which can dynamically find the cluster head and minimize the energy consumption. Thereafter, data from different sensor nodes is aggregated to the head nodes of the clusters, and data compression by principal component analysis is carried out to reduce the data transmission and cut down the energy usage. We propose an adaptive strategy of selecting the number of principal components with the compression error bound. Finally, the performances, including compression accuracy and the compression ratio, are evaluated by computer simulations, and we made a comparison with other existing PCA-based algorithms to show the effectiveness and efficiency of our proposed model.

In this paper, although we only consider the data from sensors in the monitored environment, such as temperature and humidity, the model we proposed can also be applied to other application circumstances. Our proposed model can also be extended to a multi-hop sensor network, and then, a hierarchical clustering algorithm can be used to group the sensor nodes into different clusters. Due to the fact that the relevancy among sensor nodes is obviously increased by spatial clustering, the performances of many existing algorithms will be improved. Thus, the compression mode of considering the correlation among sensor nodes can be used to enhance the performances of other existing models. Besides, more advanced works that exploit principal component analysis in a distributed way can be mentioned [28].
